# Experiences of Inuit in Canada who travel from remote settings for cancer care and impacts on decision making

**DOI:** 10.1186/s12913-021-06303-9

**Published:** 2021-04-13

**Authors:** Janet Jull, Amanda J. Sheppard, Alex Hizaka, Inuit Medical Interpreter Team, Gwen Barton, Paula Doering, Danielle Dorschner, Nancy Edgecombe, Megan Ellis, Ian D. Graham, Mara Habash, Gabrielle Jodouin, Lynn Kilabuk, Theresa Koonoo, Carolyn Roberts, Mamisarvik Healing Centre Team, Tungasuvvingat Inuit

**Affiliations:** 1 School of Rehabilitation Therapy, Faculty of Health Sciences, 31 George Street, Louise D. Acton Building, Queen’s University, Kingston, Ontario Canada; 2 Indigenous Cancer Care Unit, Ontario Health, 620 University Avenue, Toronto, Ontario Canada; 3 Mamisarvik Healing Centre, Tungasuvvingat Inuit, 25 Rosemount Avenue, Ottawa, Ontario Canada; 4 Ottawa Health Services Network Inc., 1929 Russell Road, Ottawa, Ontario Canada; 5 The Ottawa Hospital, Indigenous Cancer Program, 501 Smyth Road, Ottawa, Ontario Canada; 6 Bruyère Continuing Care, 60 Cambridge Street, North Ottawa, Ontario Canada; 7 Arctic College, Road to Apex, Iqaluit, Nunavut Canada; 8 Clinical Epidemiology Program, Ottawa Hospital Research Institute; School of Epidemiology and Public Health, University of Ottawa, 600 Peter Morand Crescent, Ottawa, Ontario Canada; 9 Larga Baffin, 2716 Richmond Road, Ottawa, Ontario Canada; 10 Department of Health, Government of Nunavut, P.O. Box 1000, Iqaluit, Nunavut Canada; 11 Tungasuvvingat Inuit, 1071 Richmond Road, Ottawa, Ontario Canada

**Keywords:** Shared decision making, Cancer care, Rural and remote, Urban, Inuit, Indigenous, Research, Co-production, Ethics, Engagement, Integrated knowledge translation

## Abstract

**Background:**

Inuit experience the highest cancer mortality rates from lung cancer in the world with increasing rates of other cancers in addition to other significant health burdens. Inuit who live in remote areas must often travel thousands of kilometers to large urban centres in southern Canada and negotiate complex and sometimes unwelcoming health care systems. There is an urgent need to improve Inuit access to and use of health care. Our study objective was to understand the experiences of Inuit in Canada who travel from a remote to an urban setting for cancer care, and the impacts on their opportunities to participate in decisions during their journey to receive cancer care.

**Methods:**

We are an interdisciplinary team of Steering Committee and researcher partners (“the team”) from Inuit-led and/or -specific organizations that span Nunavut and the Ontario cancer health systems. Guided by Inuit societal values, we used an integrated knowledge translation (KT) approach with qualitative methods. We conducted semi-structured interviews with Inuit participants and used process mapping and thematic analysis.

**Results:**

We mapped the journey to receive cancer care and related the findings of client (*n* = 8) and medical escort (*n* = 6) (“participant”) interviews in four themes: 1) It is hard to take part in decisions about getting health care; 2) No one explains the decisions you will need to make; 3) There is a duty to make decisions that support family and community; 4) The lack of knowledge impacts opportunities to engage in decision making. Participants described themselves as directed, with little or no support, and seeking opportunities to collaborate with others on the journey to receive cancer care.

**Conclusions:**

We describe the journey to receive cancer care as a “decision chain” which can be described as a series of events that lead to receiving cancer care. We identify points in the decision chain that could better prepare Inuit to participate in decisions related to their cancer care. We propose that there are opportunities to build further health care system capacity to support Inuit and enable their participation in decisions related to their cancer care while upholding and incorporating Inuit knowledge.

**Supplementary Information:**

The online version contains supplementary material available at 10.1186/s12913-021-06303-9.

## Background

Inuit are resilient. They have demonstrated self-determination and the ability to navigate and adapt to harsh and changing environments. Inuit live in many locations including urban environments, although most Inuit in Canada live in the traditional territory called Inuit Nunangat [1]. Inuit Nunangat is a region that consists of the western edge of the Northwest Territories (the Inuvialuit Settlement Region), the territory of Nunavut, northern Quebec (Nunavik), and northeastern Labrador (Nunatsiavut). Like many First Nations and Métis populations, Inuit face a high and worsening health burden in relation to others across Canada [2]. For example, Inuit experience the highest cancer mortality rate from lung cancer in the world, and the rates of morbidity for other cancers are increasing disproportionately among Inuit in relation to general populations in Canada [3, 4]. The poor health outcomes experienced by Inuit as well as First Nations, and Métis (“Indigenous”) populations in Canada are directly related to policies that undermine opportunities for Indigenous populations to engage their knowledge and address community-level needs, as stated in the Truth and Reconciliation Commission of Canada Call to Action #18 [5–7].

To obtain optimal health care, Indigenous people must negotiate complex social, historical and political systems. Inuit who live in Inuit Nunangat must travel long distances south to receive cancer and other forms of specialized health care services such as dialysis and obstetrics. They must navigate complex health systems in major urban centres often with little or no personal support. For many Inuit, decisions about accessing health care also involve decisions about commuting or moving from remote communities to a major urban centre in the south and leaving dependents, their home, employment and other community roles. The alternative is to opt out of treatment. A retrospective review of cancer treatments and outcomes among Inuit referred from Nunavut shows that, of those who are living with cancer, 70% travel to urban centres for treatment [8]. It is unclear why Nunavummiut may choose to not travel south to seek treatment, and it is not understood why women are twice as likely as men to be referred for treatment even when cancers are not sex-specific (lung and colorectal) [8].

The travel burden to access health care for those living in the northern regions of Canada has been documented [9]. For participants in our study from the Qikiqtaaluk region of Inuit Nunangat (Nunavut), the journey to receive cancer care involves the negotiation of a health system and travel over thousands of kilometres from very remote geographic areas to an urban area in Ontario. For example, the shortest travel distance to receive cancer care would be from Iqaluit, the capital of Nunavut, to Ottawa, Ontario - a distance of over 2000 km. Further, Ottawa has a population of over 1 million, which can be overwhelming for people from remote areas such as Iqaluit (population of 7000). Many Inuit live in even more remote and far smaller communities.

While all who live in remote areas of Canada are confronted with difficult decisions related to health care access, the decision making of Inuit (and other Indigenous people) are further complicated by factors related to the health care context. For example, in some regions of Canada such as Nunavut, there is limited access or a lack of organized cancer screening programs [10]. As a result, people need to have an awareness of cancer symptoms and act as a self-advocate. Additionally, they rely on a local health care system that faces perennial challenges with respect to health care provider recruitment and retention, resource constraints and high caseload challenges [11]. Finally, for Indigenous people, there is a history of negative experiences with the health care system [5] that impacts decisions to seek treatment. Inuit have painful memories about the removal of family members for tuberculosis treatment to hospitals and sanatoria located in unknown southern regions of Canada in the 1950–1960’s and from which many family members did not return [5, 7, 12]. These factors place Inuit and other Indigenous groups at risk for poor health and impose barriers to health care access.

One approach that has been found to enhance participation of Inuit in health decisions is “shared decision making” (SDM). SDM is a process by which decisions are made by the client and the health care provider(s) using the best available evidence and client preferences [13]. The client and health care provider each bring their expertise to the decision making process and may be facilitated by SDM tools and approaches such as patient decision aids and decision coaching [14, 15]. To best support Inuit to participate with health care providers in their health decisions, we identified that it was critical to understand the experiences of Inuit who travel from a remote to urban setting for cancer care, and the impacts on their opportunities to participate in decisions during the journey to receive cancer care.

There is an urgent need to improve Inuit access and use of health care and to do so in ways that promote self-determination. The United Nations Declaration on the Rights of Indigenous Peoples [16] and the Canadian Truth and Reconciliation Commission report [7] describe and promote action on the imperative for self-determination of Indigenous Peoples [17] and promote the rights of Indigenous Peoples to achieve the highest attainable health [18]. There is a need to address societal imbalances of power and support opportunities for the health and wellness of Indigenous people.

The aims of the study were to: 1) to understand the experiences of Inuit in the cancer care system, who travel from Nunavut, specifically from Qikiqtaaluk (Baffin Island) to receive their cancer care in Ottawa, Ontario; and, 2) to gain an understanding of how these experiences impact opportunities to participate in decisions during the journey to receive cancer care.

## Methods

We are an interdisciplinary team with members from Inuit-led and/or Inuit-specific organizations who are active in health care systems that provide services to communities in the Qikiqtaaluk region of Nunavut and in Ottawa, Ontario. Our team governance consists of a Steering Committee and researchers hereafter referred to as “the team”. A non-Inuit researcher team member (JJ) facilitates research activity. The work presented here is part of a larger program of research that is designed to promote Indigenous sovereignty in research [19] and in which Inuit have redefined SDM as “not deciding alone” [20]. Throughout our work, team members consult with community partners who are part of team member networks. Our aim is to is to enhance opportunities for participation of Inuit in their health care decision making and our work is described elsewhere [20].

### Research approach

The team structured the research partnership to ensure inclusion of Inuit-led and/or Inuit-specific organizations who would use or be impacted by the research (“knowledge users”) throughout the entire research study - from conceptualization of ideas through to the dissemination and application of the findings. The research approach described in our study may be referred to as “integrated knowledge translation (KT)” [21]. With integrated KT, knowledge user involvement is reflected in the approaches to study governance and the collaborative conduct of our study [22]. Our work builds on previous studies conducted in full partnership with Inuit service organizations and community member partners and uses a framework called the Collaborative Research Framework [22, 23]. As well, our research approach is aligned with the National Inuit Strategy on Research (NISR) that “outlines the coordinated actions required to improve the way Inuit Nunangat research is governed, resourced, conducted and shared” (p.3) [24] (see Additional files: Table S1).

### Guiding principles

Our team worked to conduct research that is useful and relevant for Inuit and those that support Inuit health. The team partnerships and research processes center on Inuit values and ways of knowing and are based on a foundation of the Inuit Qaujimajatuqangit guiding principles [25, 26]. The Inuit Qaujimajatuqangit principles are Inuit societal values that promote self-determination and self-reliance. They are used to serve the common good through collaborative decision making. Inuit Qaujimajatuqangit are grounded in caring for and respecting others [25, 26] and are promoted by the government of Nunavut in its initiatives and governance [27]. Throughout all phases of the research conduct, from design through to dissemination, we have reflected on the Inuit Qaujimajatuqangit principles as a guide for collaboration and knowledge development that is appropriate and beneficial for Inuit.

We conducted our study to align with views expressed by Inuit in community consultations, the Tri-Council Policy Statement-2 Chapter 9 [28] and Canadian Institutes of Health Research Guidelines for Health Research Involving Aboriginal People (2007–2010) [29]. Research ethics board approval was granted by the Nunavut Research Institute (01025 18 Registry), The Ottawa Hospital Research Institute (#20180360-01H) and Queen’s University Health Research Ethics Board (#6024694 REH-732-18) approved the conduct of the study.

### Design

We conducted the study from January to August 2019. We used qualitative research methods to uphold collaborative approaches to research and provide an effective approach to describe health care events. The use of storytelling, or ‘narrative’ methods, adds depth to the description of the health care experience [30, 31] and is an appropriate way for Inuit to share knowledge [32].

### Study setting and participants

Our study was conducted with Inuit clients and their medical escorts (“participants”) staying at Larga Baffin in Ottawa, Ontario. Larga Baffin is a full-service boarding home for residents of the region of Nunavut of Qikiqtaaluk (Baffin Island) who are Inuit beneficiaries of the Nunavut Land Claim Agreement [33, 34]. “Clients” are those who are identified by the government of Nunavut as eligible for travel to receive medical care, are residents of Nunavut, and who a health care professional has identified for medical treatment outside of the home community [35]. “Medical escorts” are defined by the government of Nunavut as people who in particular situations are recommended by a doctor or nurse to accompany a client on travel for health care, and who are approved as an escort by a regional office in Nunavut [36]. For our study, the events of interest were the experiences of Inuit who receive their cancer-related health care outside of Nunavut, and how these experiences impact opportunities to participate in decisions during the journey to receive cancer care.

### Procedure

The team developed a semi-structured interview guide, and it was pilot tested with two Inuit clients and their accompanying medical escorts, and with no amendments. The interview guide is based on frameworks that describe decisional needs [37] and previous work conducted with community members [20]. We selected semi-structured interviews to support dialogue with those in the cancer care system who travel to either receive cancer care or who support someone to receive cancer care.

The team informed potential participants of the study with a poster in English and Inuktitut to review with staff at the boarding home. Potential participants were eligible for the study if they were 18 years or older, self identified as Inuk (Inuit), able to speak English or Inuktitut, and had traveled from Nunavut to Ottawa for cancer care (to either receive cancer- or related health care, or to accompany someone as a medical escort). Boarding home contacts directed potential participants to the primary investigator (PI) (JJ) if they were interested in learning more about the study. As the PI was not able to communicate in Inuktitut, potential participants were invited to have an interpreter present to support the review of the consent forms and/or to be present for interpretation during the interview. The participants reviewed the consent forms and then provided written or verbal consent to participate in the study. Then, the PI interviewed participants for 20–45 min, which included a request for non-identifying demographic information. During interviews, the PI invited participants to describe the experiences that led to their receiving cancer care in the urban setting. The PI invited participants to describe how these experiences may have had an impact on their opportunities to participate in decisions during their journey to receive cancer care. The interviews were digitally recorded (*n* = 13) or recorded in notes taken by the PI (n = 1) according to participant preferences. If needed, interviews were translated into English (*n* = 4). We had the interviews transcribed verbatim. The PI removed participant names and identifying characteristics to preserve anonymity.

### Analysis

The PI analyzed the transcribed interviews using the methods of process mapping [38] and thematic analysis [39]. The team members acted as secondary reviewers to confirm the identified themes. We used process mapping [38] adapted for people who are using the health care system [40]. For each interview, the PI took participant reports of places and events that were de-identified, organized them in a chronological order, and then reported these as a collective summary map of the journey to receive cancer care. The team had the process explained and reflected to them by the PI, and the entire team co-developed and confirmed the summary map. The aim was to map the journey from the perspective of participants, starting with the entry into the health care system from the home community and moving through to the delivery of cancer care in the urban setting. We mapped the journey from the orientation of systems’ users to explain the events that led up to the receipt of cancer care, to understand where opportunities to participate in decisions may exist.

We also conducted thematic analysis of participant interviews. The PI analyzed transcribed interviews using a six-phase process of thematic analysis [39]. The process was iterative and used the steps of 1) familiarization with data; 2) generation of initial codes within each transcript; 3) search for themes; 4) review of themes; 5) define and name themes, which were further confirmed or adjusted by second reviewers (team members); and 6) reporting of themes [39]. The PI was central to the process and the findings reflected to the team for confirmation. The team agreed on the final findings. The approach to data analysis supported meaningful understandings of how events may impact opportunities to participate in decisions during the journey to receive cancer care.

## Results

### Participants

Fourteen people who identified as a client (*n* = 8) or a medical escort (*n* = 6) participated in the study. Participants self-identified as Inuit, between the ages of 20 and 80 years and most indicated that they are responsible for the care of children, Elders, or extended family and/or community members. More participants self-identified as male (*n* = 9) than female (*n* = 5). Most participants reported that they had made six or more trips for cancer care, with some indicating that they had made the trip a few times or that it was their first time. Most participants reported being away for more than one month, with a few indicating that they would be away a week or two, or unsure for how long they would be away. We have reported the identifying characteristics of the study participants in a way that preserves their anonymity.

**Fig. 1:**
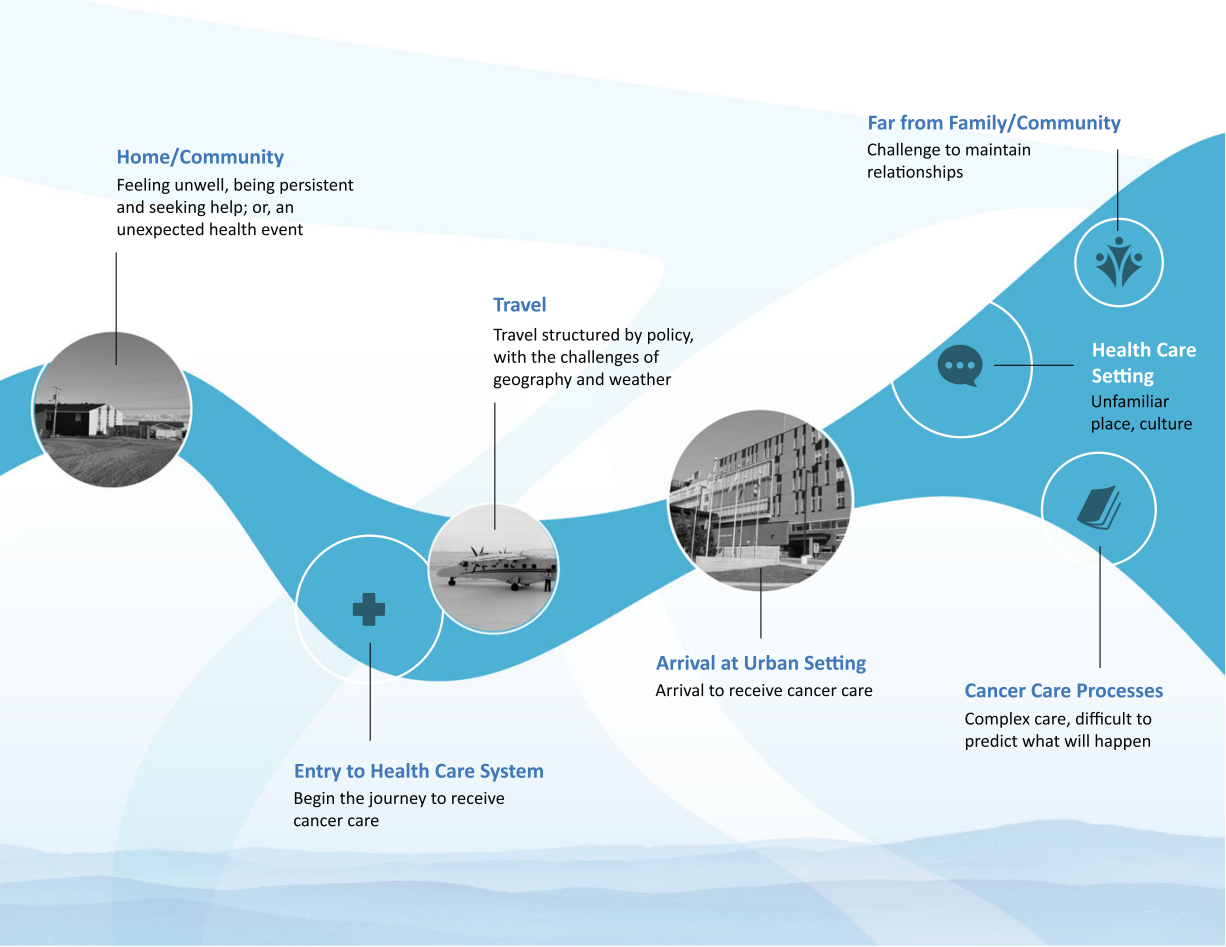
Map: The journey to receive cancer care

We identified four major themes about the participant experience of the journey to receive cancer care and how these experiences may impact opportunities to participate in decisions. We report the four themes in relation to the map of the journey to receive cancer care to explain the events around three main events as follows: at the initial entry into the cancer care system, travel, and arrival to receive cancer care services. (Fig. 1)

(see Additional files, Details on themes).

### Theme #1: it is hard to take part in decisions about getting health care

***“It takes forever to try and get what we want to say to the doctors”.***

The first theme relates to the experience of clients and medical escorts at the start of their journey to receive cancer care and is depicted on the map as starting at the home community and with entry into the health care system. (Fig. 1). The clients described either: feeling unwell and being persistent in seeking help; or, having no idea of their health status until receiving care for other issues (such as an acute illness, or injury) that led to their initial diagnosis and entry into the cancer care system. The medical escort participants reported a sudden request to accompany a client out of the community. Clients described having to be persistent to get attention for worrying health issues. Some clients described having worsening bowel issues (e.g. blood in stool, increasing abdominal pain) that community health care providers had dismissed on numerous health centre visits. These clients described how, when sent on medical travel outside of the community to see a physician specialist about another unrelated issue, they took the opportunity to explain their health issues. These clients described how the health care provider then initiated tests that were eventually followed by a cancer diagnosis: “Since we don’t have any doctors up in each community, it takes forever to try and get what we want to say to the doctors. So when I went down in November to Iqaluit [for a reason unrelated to the symptoms], the doctor there said you have to be checked about your bottom. That’s when I found out I had cancer. But I was trying to tell them before”. Most of the medical escorts reported receiving a call from the medical travel clerk telling them to accompany a client outside of the community. All of the medical escorts described their engagement as sudden: “My [partner] was unwell … [name] went back and forth to health centre many times - I think - six or more? Finally [name] saw the doctor, who referred out of community. I only knew something was happening when I had a call from medical travel.” All participants related their initial entry into the cancer care system as very sudden and with limited or no opportunities to participate in decisions about obtaining their health care. In other words, they were directed as to what to do. The result is that people did not enter the system as active partners in the journey to receive cancer care.

### Theme #2: no one explains the decisions you will need to make

***“They didn’t explain anything, they just said you’re going to Ottawa to see a doctor.”***

The next theme depicted on the map is travel. The journey to receive cancer care involves travel structured by policy and complicated by the challenges of geography and weather. Clients and medical escorts must travel thousands of kilometres for cancer care that often involves more than one flight (Fig. 1). The participants in our study were unclear about the medical travel policy that structures the delivery of their health care. Clients described wanting to understand and participate in their cancer care. One client described how having no idea of what would happen led to anxiety: “It wasn’t really easy, coming down. What am I coming into? You know what I mean? It was a mystery”. The lack of understanding extended to when a medical escort is permitted to accompany a client or for how long a medical escort may stay to assist a client. The medical escorts who identified themselves as new to their role and the cancer care system, related that they faced challenges to understand how to best support clients. One medical escort described how they received a phone call from a medical travel clerk while at work and was told to be at the airport in a few hours to accompany a relative on a flight out of the community. The medical escort explained that as they wanted their relative to get the health care, they had to walk off a job to be at the airport on time: “My supervisor [name] is a good person and understood why I had to go”. The medical escorts described how they learn from experience about how to support the client they accompany on the journey to receive cancer care.

Some participants had previous experience and talked about how they have learned to navigate the health care system**.** One client who had accumulated experience over many months of cancer treatment described some clinic appointments as taking a long time to get. The client explained that it is important to be active and to manage these appointments in relation to long travel distances and the uncertain weather. The client also described how to intervene and avoid medical travel scheduling errors: “When we go home [back to the community from Ottawa], our doctor makes appointments [for the next visit]...sometimes back north transportation people make reservations on the appointment day [in Ottawa]. It hasn’t really gotten to me, it almost did, but I’m a talker and they fixed it right away...They were sending me [to travel] on my appointment day and I asked why. And they said, I don’t know, we’ll check”. These clients described themselves as learning from accumulated experience about how to participate in decisions during their journey to receive cancer care. Similarly, the medical escorts who had previous experience understood and knew they could engage in decisions with those facilitating medical travel: “[we] can talk directly to the people at OHSNI [Ottawa Health Services Network Inc.] ahead of time and then they can cooperate with the health center and make suggestions [for travel arrangements]. There would be no one to turn to down here if there wasn’t OHSNI, right? Come to think of it”. The medical escorts who had learned from experience described their strategies to participate and support the client in decisions during the journey to receive cancer care. Participants also identified the geography and weather as complicating factors in the decisions about what will happen in their cancer care, and described by a client: “… sometimes, because of the weather, they [health care providers] want to make sure I won’t get stuck up there. They were worried about that last time I came down. They’re worried I’m going to get stuck, because they don’t have all that hospice stuff back home. Chemotherapy, you can’t do that up north”. All participants agreed that the journey to receive cancer care is complex due to medical travel policy, geography, and weather. Those participants who had learned from previous experience described being able to anticipate and participate in decisions on the journey to receive cancer care.

### Theme #3: there is a duty to make decisions that support family and community

***“It is important that they are not alone”.***

The next theme depicted on the map relates to the experiences of participants on arrival at the urban setting to receive cancer care and the impacts on their participation in decisions as they navigate the urban setting, described as: 1) far from family/community, 2) in a health care setting, and 3) engage in cancer care processes (Fig. 1). In our study, participants described how they worked to find ways to collaborate with others in the journey to receive cancer care: “That’s our life, sharing”. Participants described strategies to work with family and community members to negotiate decisions about receiving cancer care in the urban setting and the importance of support to navigate decisions related to cancer care processes and the impacts on medical escorts who accompany clients.

Participants described how strategies, such as the use of technology, can support them to manage complicated family arrangements. This was explained by a client: “My daughter is pregnant and she had to go to [city] [due to the medical evacuation policy] until the due date, and my 13-year old daughter had to follow her sister. Because I didn’t have a sitter right away, in my hometown …. Nowadays these [cellphones] are good”. Another client explained how previous life experience had made them aware of the impacts of a family member’s absence and the importance of maintaining contact: “… when I was a kid, my Dad was away for six years in hospital and there was no communication, no telephone, we never – my Mom, myself – heard from him for so long”. The clients also talked about how they rely on support from the medical escorts to help them navigate decisions in their cancer care while in the urban setting. One client explained the importance of support: “If I don’t make a decision, I’ll talk to [medical escort] a little bit and [medical escort] lets me decide”. These client experiences highlight the important role of medical escorts and other supports in negotiating the many day-to-day decisions required to successfully navigate and receive cancer care. For, as one client explained: “I could not have done it by myself, without help”.

Clients in our study also reported appreciation for the support from medical escorts who had decided to accompany them on their journey to receive cancer care. Some clients expressed concern about the impacts on medical escorts: “But yesterday … it was very stressful and tiring. It’s difficult for [medical escort] to be here. I think [medical escort] should be living in housing, for [them] to be here as escort, they’re not making any money for it. They’re not working, so they’re losing money. I think there should be some compensation for that”. When asked directly about the impacts of the decision to be a medical escort, medical escort participants explained that health care for relatives and community members is a priority: “… I’ve got a 7-year-old to look after and last year I was away for the whole year. That was uncomfortable for me because I have to look after [child], because he gets [describes a medical issue]. But I don’t mind if we have to come down to Ottawa, I don’t mind following my [relative], because nobody … wants to follow [relative]”. The experiences of participants who are in the urban setting for their cancer care centred on how it is critical that clients receive support and have opportunities to collaborate with others and participate in decisions that make it possible to receive cancer care.

### Theme #4: the lack of knowledge impacts opportunities to engage in decision making

***“I didn’t have a clue about what was going to be happening”.***

The final theme relates across the entire journey to receive cancer care, depicted on the map as starting in the home community and through to the urban setting (Fig. 1). Participants described the lack of knowledge about cancer care as having an impact on their opportunities to engage in decisions throughout the journey to receive cancer care. Some clients explained that they had tried to address their limited understandings of how the cancer care system works as a way to participate in decisions about their cancer care: “When I ask back home, who’s making all these decisions that I have to go to this, or they’re saying you have to be in Ottawa this certain time if I like it or not, even if it interferes with something. And they just tell me, it’s not us, it’s ‘Ottawa’ “. In addition, clients also described the lack of knowledge and the stress of negotiating the cancer care system as leaving them unprepared to participate with health care providers who provide cancer care: “They explain good but when they ask me “any questions?“ my mind just gets blank. I don’t know why”. Medical escorts without experience of the cancer care system also described the lack of knowledge as a barrier in their role to support clients. A medical escort described how there was no information about what would happen before they set off from the community to accompany the client to the urban setting for cancer care: “They didn’t explain anything. All they said is you’re going to Ottawa, you have to go” All participants described a lack of knowledge about the cancer care system as a factor that undermines opportunities for Inuit to participate in decisions during the journey to receive cancer care.

## Discussion

Our study relates the experiences of Inuit clients and medical escorts who travel from the remote areas of Qikiqtaaluk region of Nunavut to the urban setting of Ottawa, Ontario for cancer care.

Our study findings show that the journey to receive cancer care consists of a series of connected events that we describe as a “decision chain”. (Fig. 1). Participants described themselves as directed, with little or no support, and seeking opportunities to collaborate with others on the journey to receive cancer care. We identify key points in the decision chain where there are opportunities to build system capacity to prepare people to participate in decisions related to their cancer care.

### Support on the journey to receive cancer care

The participants in our study described a range of challenging situations that they and their families face and the importance of support during their journey to receive cancer care. People who live in remote areas are identified as being at risk to experience stress as, to receive cancer care, they must leave their family and community supports to travel to the location of care [41]. Research with Indigenous populations who live in remote areas shows that health care systems do not accommodate the context and logistic complexity of health care access [42–44]. Inuit have been identified to be at increased risk of harms during their transition to urban centres [45], including travel for medical care [46]. The recommendations of The Missing and Murdered Indigenous Women and Girls Inquiry (MMIWG) call on governments to plan and fund safe, sufficient and readily available transportation in towns and cities with particular consideration of the limited transportation available, especially in fly-in, northern and remote locations [47, 48]. Our study identifies that Inuit experience social and financial burdens in relation to the journey to receive cancer care, a finding that is documented elsewhere [49]. Inuit and others in rural and remote areas need support to safely make the journey to receive cancer care.

Inuit need to know that they are not alone and have support on their journey to receive cancer care. In studies conducted with general populations about the journey to receive cancer care, the importance of support has been highlighted [47]. In a study with Aboriginal Australians, interpersonal supports were found to make it possible for clients to better negotiate system and health care provider barriers to care [43]. The health care system needs to be structured to support client, medical escorts and their families and community members to collaborate with health care providers and others who facilitate or deliver health care.

### Opportunity to collaborate with others on the journey to receive cancer care

Every cancer journey is unique and so is unpredictable. As well, cancer care is complex and often delivered by a team of health care specialists. Studies with general populations identify that clients often feel they are not active participants in developing their cancer treatment plans despite wanting to communicate with their health care team about what matters to them and to have their preferences incorporated into their care plans [41, 50–52]. Here, we have related the experiences of Inuit clients and their families at each stage of the journey to receive cancer care. The participants with previous experience in the journey to receive cancer care described how they have independently found ways to collaborate with others in the health care system and to negotiate the details of their cancer care; however, these strategies may not be available to others without previous experience in the cancer care system. As well, these strategies may not be inclusive of Inuit knowledge and so may not be perceived as useful to all Inuit.

In our previous work, we have identified that many Inuit are not prepared to participate in conversations with health care providers when they arrive in the urban setting for their health care [20]. When Inuit arrive in the urban setting to meet with health care providers, it is often without any opportunities to participate in decisions or knowledge about what is happening. Inuit partners in our work and participants in other studies report that it is overwhelming to then be asked to participate in health care decisions [20], a finding reported elsewhere with Indigenous clients [41]. Studies show that with general populations, people with serious illness often feel unable to share in decision making with health care providers [53]. Client and health care provider participants identify cancer care systems as not operating in a shared care model. Instead, clients are left to coordinate their care and those who must travel from rural and remote areas face additional challenges [41]. People need to have opportunities to collaborate with others and to be prepared to engage in decisions on their journey to receive cancer care.

Research shows that to be able to play an active role in decision-making with their health care providers, clients need to be informed and empowered [54]. “Patient activation” refers to clients’ assessment of their understanding, confidence and readiness to manage their own health [55]. While patient activation has been linked with positive health outcomes, it may be an important precursor to prepare for and participate in SDM with health care providers [56]. In general populations, SDM has been found to improve clinical decision-making processes [57], client satisfaction with health services [58] and client health experiences and outcomes [55, 59]. SDM is of particular benefit to those who face adversity in the health care system [60]. Our research supports the use of SDM by Indigenous populations to better participate with health care providers [20, 61–63], with a particular focus on preparation for participation in SDM that is founded on Inuit knowledge [19, 20]. Inuit research partners have identified that working together to make decisions about health upholds Inuit Quajimajatuqangit principles in health care systems [20] and, in particular, ***Aajiiqatigiinniq:*** Decision making through discussion and consensus [27].

We propose that there are opportunities to build capacity in the cancer care system to support Inuit to collaborate with others in their journey to receive cancer care. We identify key points in the decision chain to better support Inuit to be prepared to participate in decisions related to their cancer care. We identify *what is currently done* to engage Inuit with those who facilitate or deliver health care services. Further, we examine *what could be done* to better support Inuit and enhance their opportunities for participation in their cancer care. We use an example to describe how to build health care system capacity to support Inuit to participate in decisions related to their cancer care in a way that upholds and incorporates Inuit knowledge [65–75] (see Additional files: Table S2).

### Limitations and strengths

One limitation of our study is that we engaged a small group of participants who are from a particular region of Inuit Nunangat and who were based, at the time of the study, in an urban setting (Ottawa). Depending on the location of their home in Inuit Nunangat, Inuit might travel to very different urban settings for health care, each with unique provincial health care delivery systems and supports. As well, many community members met with the researchers to learn about and discuss the study but were reluctant to engage formally in the research. Our study may have possibly included participants who were more used to the Western-oriented approach to research. The strengths of the present study are that we used an integrated KT approach to build and foster relationships of an interdisciplinary team that include members of the Inuit community and that we sustained throughout the entire research study. As well, our approach to research ensures that the views of those who work in cancer care systems and provide care to Inuit clients are included so that our work is more likely to create knowledge that is useful and relevant [21]. We used Guba and Lincoln’s (1982) criteria for trustworthiness in qualitative research known as credibility, dependability, confirmability and transferability [64].

(see Additional files: Table S3).

## Conclusions

Our study relates the experiences of Inuit clients and medical escorts who travel from remote areas of Nunavut to an urban setting in Ontario for cancer care. We report on how these experiences impact opportunities to participate in decisions during the journey to receive cancer care. Participant experiences show that Inuit are often unprepared to participate in the series of health systems’ events they must navigate on the journey to receive cancer care. We describe the series of events as a “decision chain” that extends throughout the journey to receive cancer care. Participants describe themselves as directed with little or no support and seeking opportunities to collaborate with others on the journey to receive cancer care. We identify key points in the decision chain to prepare Inuit to participate in decisions related to their cancer care. We propose that there are opportunities to build capacity in the health care system to support Inuit to participate in decisions related to their cancer care, and that uphold and incorporate Inuit knowledge.

## Supplementary Information

**Additional file 1 Table 1.** The five priority areas for research identified by and committed by Inuit Tapiriit Kanatami (ITK) in the National Inuit Strategy on Research [24], and research actions in our study.

**Additional file 2 Table 2.** Decision chain events.

**Additional file 3 Table 3.** Reporting Criteria for Trustworthiness [64].

**Additional file 4.** Details on themes.

## Data Availability

Supporting data may be requested from the corresponding author on reasonable request.

## Abbreviations

SDM: Shared decision making
PI: Principle investigator
OHSNI: Ottawa Health Services Network Inc.
NISR: National Inuit Strategy on Research
MMIWG: Missing and Murdered Indigenous Women and Girls
