# Supplementary material for: Experiences of Inuit in Canada who travel from remote settings for cancer care and impacts on decision making
Source: BMC Health Serv Res. 2021 Apr 13;21:328. doi: 10.1186/s12913-021-06303-9 (PMC8042963; doi:10.1186/s12913-021-06303-9)
Supplement: Supplementary file 1 — Additional file 1 Table 1. The five priority areas for research identified by and committed by Inuit Tapiriit Kanatami (ITK) in the National Inuit Strategy on Research [24], and research actions in our study. [file 12913_2021_6303_MOESM1_ESM.docx]

**Additional files: Table 1.** The five priority areas for research identified by and committed by Inuit Tapiriit Kanatami (ITK) in the National Inuit Strategy on Research (24), and research actions in our study.

| Priority areas for research | Research actions in our study |
| --- | --- |
| 1) Advance Inuit governance in research | Study governance and conduct structured as partnerships between researcher and Inuit community and organizations. The foundations of the work are in the IQ principles and guidance of Inuit members. |
| 2) Enhance the ethical conduct of research | Development and conduct reflect the guidance of the National Inuit Strategy on Research (24) as well as other key guidance documents for Indigenous health (29, 65, 66). |
| 3) Align funding with Inuit research priorities | Study developed in response to Inuit community and organizational priorities. Steering community members consist of Inuit community and organizational members. |
| 4) Ensure Inuit access, ownership, and control over data and information | Recognize Inuit community and organizations as partners in the research; participation in development of study protocol and involved for data collection, interpretation and dissemination; data stored in mutually-agreed upon ways that protects privacy and confidentiality of participants; researcher-Inuit community and organizational partners behave in a collaborative manner (e.g. create opportunities for meetings, informed questions about study procedure, email and in-person contact for dialogue and agreements about how and who to disseminate knowledge in mutually-agreed upon ways) (24). |
| 5) Build capacity in Inuit Nunangat research | Full partnership with members of Inuit Nunangat society as team members; building relationships with institutions located in Inuit Nunangat, for their research leadership in current and future steps of the research process. |
