# Supplementary material for: Experiences of Inuit in Canada who travel from remote settings for cancer care and impacts on decision making
Source: BMC Health Serv Res. 2021 Apr 13;21:328. doi: 10.1186/s12913-021-06303-9 (PMC8042963; doi:10.1186/s12913-021-06303-9)
Supplement: Supplementary file 2 — Additional file 2 Table 2. Decision chain events. [file 12913_2021_6303_MOESM2_ESM.docx]

**Additional files: Table 2.** Decision chain events

| **Decision, event** | **What is currently done to engage clients and those who facilitate or deliver services in the health care system** | **What could be done to engage clients in the health care system, with examples** | **How event links to shared decision making (SDM)** |
| --- | --- | --- | --- |
| **Home/community:** decisions about entry to the cancer care system. | | | |
| The journey to receive cancer care begins through client persistence, or an unexpected event. | Community health centres (Western-oriented) provide health care in communities:  Nunavut’s 25 communities receive health services through 22 community health centres staffed by nurse practitioners and registered nurses; physicians and some specialists visit health centres and provide advice by telephone;  There are regional health centres in Rankin Inlet and Cambridge Bay, and the Qikiqtani General Hospital in Iqaluit and have physicians available full-time;  There is chronic shortage of health care providers in Nunavut (67). | Facilitation of relationships to allow community members and health care providers to navigate the health care system, together.  Community health representatives (CHRs) bridge community life with the health care system, and their job is to work with health professionals in the prevention of disease and maintenance of health (<https://www.gov.nu.ca/community-health-representative>)  We have been developing a strategy for use by community health providers, such as CHRs, to support preparation for SDM process with Inuit (19, 20).  Community members could be prepared to engage in conversations about their care. For example,  a church-based intervention to promote informed decision-making for prostate cancer screening among African-American men aimed to increase knowledge about the benefits, risks and limitations of prostate cancer screening; and promote confidence in men’s ability to participate in the decision making process at a level that is personally desired (68).  Health care providers who are not from the community can be prepared to engage with and support community members in decisions about their health care. For example, a mobile dialysis bus was found to benefit Indigenous clients’ social and emotional wellbeing and to provide a valuable cultural learning opportunity for staff (69). | To be ready to participate in SDM, people need to know that it is their right to have their say in decisions about their health.  Community member/client and health care provider activation in health care partnerships are an important feature of preparing to engage in SDM (55).  Inuit societal values promote working together for a common cause (25, 27). |
| **Travel**: decisions related to negotiation of cancer care system, such as medical travel plans, medical escort. | | | |
| The entry to the health care system begins the journey to receive cancer care. Clients and medical escorts must negotiate travel that is structured by policy with the challenges of geography and weather. | Clients receive a telephone call from a contact at medical travel.  Under particular circumstances, clients who must travel for medical care may be eligible to have an escort travel with them and to accompany them during their medical care (36). | Explanations of medical travel policy can help people to understand the complex system, for example using plain language (70) and posting information in places where people who do not have computers can access the information.  Resources that orient clients and medical escorts to the health care system can be used to prepare people to collaborate and negotiate their journey to receive cancer care, for example:  Nunavut Navigation  <https://www.youtube.com/watch?v=Ny5t-ZlRGpM&feature=youtu.be>  Chemotherapy Navigational Video  [https://youtu.be/iJnMiDYfC-8](https://can01.safelinks.protection.outlook.com/?url=https%3A%2F%2Fyoutu.be%2FiJnMiDYfC-8&data=02%7C01%7Cjanet.jull%40queensu.ca%7C7c54f5ced9a74a86cfc508d74765ec7c%7Cd61ecb3b38b142d582c4efb2838b925c%7C1%7C0%7C637056376080468105&sdata=o4n7N2TwVUMygOE%2FVFR1gJUCPuNm5%2F2w0rfmOwsPbvg%3D&reserved=0) (Inuktitut)  [https://youtu.be/fzXzsdjSEaU](https://can01.safelinks.protection.outlook.com/?url=https%3A%2F%2Fyoutu.be%2FfzXzsdjSEaU&data=02%7C01%7Cjanet.jull%40queensu.ca%7C7c54f5ced9a74a86cfc508d74765ec7c%7Cd61ecb3b38b142d582c4efb2838b925c%7C1%7C0%7C637056376080478101&sdata=Gkv30nIyvAXeVE1SVQmGKr%2B7hBXCKpHfbxHZbGGa%2B2Q%3D&reserved=0) (English)  Radiation Therapy Navigational Video  [https://youtu.be/aB-iLOpW86w](https://can01.safelinks.protection.outlook.com/?url=https%3A%2F%2Fyoutu.be%2FaB-iLOpW86w&data=02%7C01%7Cjanet.jull%40queensu.ca%7C7c54f5ced9a74a86cfc508d74765ec7c%7Cd61ecb3b38b142d582c4efb2838b925c%7C1%7C0%7C637056376080478101&sdata=csdm3DNwItpG3fobGIPxDun6klJc6HYNyqCgKd5wWe8%3D&reserved=0) (English)  [https://youtu.be/oaQoec-BpS8](https://can01.safelinks.protection.outlook.com/?url=https%3A%2F%2Fyoutu.be%2FoaQoec-BpS8&data=02%7C01%7Cjanet.jull%40queensu.ca%7C7c54f5ced9a74a86cfc508d74765ec7c%7Cd61ecb3b38b142d582c4efb2838b925c%7C1%7C0%7C637056376080488091&sdata=FWdxIB%2FAEoxYHEj08g25bNHUW5zXTDfkxj0PnnZeBpA%3D&reserved=0) (Inuktitut) | When information is shared in ways that are meaningful to recipients, the information supports self-efficacy for clients (71).  It is important to support self-efficacy, as Inuit societal values uphold respect for self-determination, through being innovative and resourceful, and development of skills (25, 27). |
| **Urban setting**: decisions related to receipt of cancer care. | | | |
| Arrival at the urban setting to receive cancer care. | Clients and their medical escorts may face challenges when arriving in the urban setting for health care, and for which there are some supports in place (33, 72, 73):  -they are far from family/community and must find ways to maintain these important relationships  -the health care setting may be very different to what they are used to, both the place and culture  -the cancer care processes are complex and are delivered in a Western-oriented manner (versus Inuit). | SDM tools/approaches can be used to support clients to participate with urban-based health care providers and make high quality health decisions, in ways that are informed and consistent with client preferences.  Inuit-led and oriented services can be further utilized to support and integrate client preferences and values into the delivery of health care. The use of supports and services that are culturally resonant with Inuit clients, such as those provided at the medical boarding home and by the interpreter team, help Inuit to negotiate cancer care in the urban setting (33, 72).  For example, in a study designed to evaluate the effects of training physicians in SDM, it was found that when health care providers use SDM tools and strategies patients can be more involved in treatment decisions, and the risks and benefits of treatment options can be explained in more detail without adversely affecting patient-based outcomes (74). | SDM occurs with the mutual expertise of health care providers and client views and values.  It is important to support clients to work together with health care providers to make what the client can define as a high quality decision.  A high quality decision is:  informed, consistent with client preferences, acted upon (75), and considers the relational features of the decision. That is, the approach to decision-making includes consideration of who will be impacted by the decision.  In our program of study called “Not Deciding Alone”, the best decision is one that the client defines as best for themselves and family, community. The relational approach to making health decisions is reflects the Inuit approach to collective decision-making (20).  Inuit societal values focus on respecting others, relationships and caring for people; and serve and provide for family and/or community (25, 27). |
