# Supplementary material for: Experiences of Inuit in Canada who travel from remote settings for cancer care and impacts on decision making
Source: BMC Health Serv Res. 2021 Apr 13;21:328. doi: 10.1186/s12913-021-06303-9 (PMC8042963; doi:10.1186/s12913-021-06303-9)
Supplement: Supplementary file 3 — Additional file 3 Table 3. Reporting Criteria for Trustworthiness [64]. [file 12913_2021_6303_MOESM3_ESM.docx]

**Additional files: Table 3.** Reporting Criteria for Trustworthiness (64)

| Criteria | Study Action |
| --- | --- |
| To establish credibility, that is, the confidence that the results from the perspective of the participants are true, credible and believable: | The interviewer (JJ) spent prolonged time in the interview setting (as a volunteer for over a year) and worked with the support of community members; the interview guide was tested with volunteer community members, and there were regular meetings with the steering committee during the study conduct. |
| To establish dependability, and ensure the findings of the qualitative study are repeatable if it were to occur within the same cohort of participants, coders, and context: | We have reported on the study conduct in detail with rich description of the study methods and developed an audit trail for all steps of the study. |
| To establish confirmability and extend the confidence that the results would be confirmed or corroborated by other researchers. | We engaged in regular meetings about the study conduct; we provided dense description of research methods, and triangulation of evidence from a variety of sources (the team and networks, community members). |
| To establish transferability, the degree to which the results can be generalized or transferred to other contexts or settings. | We conducted purposeful sampling and reached data saturation on themes. |
