# Supplementary material for: Experiences of Inuit in Canada who travel from remote settings for cancer care and impacts on decision making
Source: BMC Health Serv Res. 2021 Apr 13;21:328. doi: 10.1186/s12913-021-06303-9 (PMC8042963; doi:10.1186/s12913-021-06303-9)
Supplement: Supplementary file 4 — Additional file 4. Details on themes. [file 12913_2021_6303_MOESM4_ESM.docx]

Additional files: **Details on themes**

**Theme #1: It is hard to take part in decisions about getting health care.**

***“It takes forever to try and get what we want to say to the doctors”***

The first theme relates to the experience of clients and medical escorts at the start of their journey to receive cancer care and is depicted on the map as starting at the home community and with entry into the cancer care system. (Figure 1). The clients described either feeling unwell and being persistent in seeking help; or, having no idea of their health status until receiving care for other issues (such as an acute illness, or injury) that led to their entry into the cancer system. The medical escort participants reported a sudden request to accompany a client out of the community.

Clients described having to be persistent to get attention for worrying health issues. Some clients described having worsening bowel issues (for example, blood in stool, increasing abdominal pain) that community health care providers had dismissed on numerous health centre visits. These clients described how, when sent on medical travel outside of the community to see a physician specialist about another unrelated issue, they took the opportunity to explain their health issues. These clients described how the health care provider then initiated tests that were eventually followed by a cancer diagnosis. One client described what happened: “Since we don't have any doctors up in each community, it takes forever to try and get what we want to say to the doctors. So when I went down in November to Iqaluit [for a reason unrelated to the symptoms], the doctor there said you have to be checked about your bottom. That's when I found out I had cancer. But I was trying to tell them before".

Another client talked about how they had to be persistent and advocate for themselves about their health: “[I] had gone back and forth to health centre for months - more than 3 times - due to weakness, bloating, pain. [I was] sent home with Tylenol by healthcare providers. Then, [I] had test on a visit for something else, and [I was] sent to urban centre for further investigation. I found out I had [cancer]”. The clients described their entry into the cancer system as sudden, and with no opportunity to participate in decisions about their entry into the cancer care system. One client described being asked told to wait at the health centre for a flight out of the community to travel thousands of kilometers to an urban centre: “I had no money, clothes, no chance to tell my family”.

A few of the clients reported that they had no idea that they might be ill prior to receiving their cancer diagnosis. These clients described being launched into the cancer care system without any opportunity to anticipate or be active in decisions. For example, one client described how they had been hurt in an accident that led to medical evacuation to Ottawa. The client described receiving a cancer diagnosis following what they had understood to have been routine bloodwork in their injury treatment: "I had no idea I was even sick until I [was injured] and got medevac’d down here. And then I found out I had cancer in my lung. I had an operation and they took it out, but it's [spread to other areas]”. Another client described being unwell with an “…illness that caused symptoms, [I] had to go to Ottawa for "a check up". When I came up here to this boarding house, that's when I found out I have lung cancer. That's it….it was a surprise, yes".

Most of the medical escorts reported receiving a call from the medical travel clerk telling them to accompany a client outside of the community. All of the medical escorts described their engagement as sudden: “My [partner] was unwell…[name] went back and forth to health centre many times - I think - 6 or more? Finally [name] saw the doctor, who referred out of community. I only knew something was happening when I had a call from medical travel.” Some of the medical escorts in our study said they were asked to be medical escorts by the immediate family members of the client. One participant explained how the clients’ family was unable to take on the role of medical escort: “I was asked by clients’ supports to be medical escort, as I’ve….done [medical travel for cancer care] before and they saw I managed well”. Another participant explained how who would play the medical escort role was negotiated within the family, due to jobs, family support: "The person I escort lost [their] friend, and kids, [they] are busy - and the family agreed"

All participants related their entry into the cancer care system as very sudden and with limited or no opportunities to participate in decisions about obtaining their health care; that is, they were directed in what to do. The result is that people do not enter the system as active partners in the journey to receive cancer care.

**Theme #2: No one explains the decisions you will need to make in the journey to receive cancer care:**

***“They didn’t explain anything, they just said you’re going to Ottawa to see a doctor.”***

The next theme depicted on the map is travel: the journey to receive cancer care involves travel structured by policy with the challenges of geography and weather. Clients and medical escorts must travel thousands of kilometres for cancer care, and that often involve more than one flight (Figure 1).

The participants in our study were unclear about the medical travel policy that structures the delivery of their health care. A client described the challenges of navigating a system without understanding of the medical travel processes: "…they give me a piece of paper with a schedule of appointments and say you have to be down here about a day or two before my first appointment. But when I start to talk with somebody about that [medical travel] they say, we don't make the schedules, Ottawa makes the schedules [clinic schedules]. You have to go down there whether you like it or not. I say Okay, that's not making it easy for me, especially if I have to stay [in Ottawa] for up to three months and housing up there [Nunavut], they have rules for tenants [residents will lose their housing if they are absent for a certain length of time]. So I have to make sure I can go home after six weeks or 70 days, and get it all straightened out”.

One client described wanting to understand and participate in their cancer care. The client described how having no idea of what would happen led to anxiety: “"It wasn’t really easy, coming down. What am I coming into? You know what I mean? It was a mystery”. The lack of understanding extended to when a medical escort is permitted to accompany a client or for how long a medical escort may stay to assist a client. The medical escorts who identified themselves as new to their role and the cancer care system, related that they faced challenges to understand how to best support clients. One medical escort described how they received a phone call from a medical travel clerk while at work and was told to be at airport in few hours to accompany a relative on a flight out of the community. The medical escort explained that as they wanted their relative to get the health care, they had to walk off a job to be at the airport on time: “My supervisor [name] is a good person, and understood why I had to go”.

Medical escorts described the experience of uncertainty about what would happen on the journey to receive cancer care. They also talked about how they wanted to help the client get their cancer care: "The first time - I did not learn how long I would be here. I had a call from referral and was told which airline to be at that day… [on the airplane, the client] was seated way in back, I was way in front and had to keep looking. Ask him, what if he needs something - anything". Another medical escort described how they had no information about how to support the client on the journey to Ottawa: "They didn’t explain anything. All they said is you’re going to Ottawa, you have to go. They never explained that I had to go down to Ottawa to escort [client]…you knew it was a health care thing that was all you knew” The medical escorts described how they learn from experience about how to support the client they accompany on the journey to receive cancer care.

Some participants had previous experience and talked about how they have learned to navigate the health care system**.** One client who had accumulated experience over many months of cancer treatment described some clinic appointments as taking a long time to get. The client explained that it is important to be active and to manage these appointments in relation to long travel distances and the uncertain weather. The client also described how to intervene and avoid medical travel scheduling errors: "When we go home [back to the community from Ottawa], our doctor makes appointments [for the next visit]...sometimes back North transportation people make reservations on the appointment day [in Ottawa]. It hasn’t really gotten to me, it almost did, but I'm a talker and they fixed it right away...They were sending me [to travel] on my appointment day and I asked why. And they said, I don't know, we'll check". These clients described themselves as learning from experience about how to participate in decisions during their journey to receive cancer care.

Similarly, the medical escorts who had previous experience understood and knew they could engage in decisions with those facilitating medical travel: “[we] can talk directly to the people at OHSNI [Ottawa Health Services Network Inc.] ahead of time and then they can cooperate with the health center and make suggestions [for travel arrangements]. There would be no one to turn to down here if there wasn’t OHSNI, right? Come to think of it”. The medical escorts who had learned from experience described their strategies to participate and support the client in decisions during the journey to receive cancer care.

Many participants alluded to the complications of travel from remote areas, and weather that caused delays or sudden changes in their travel plans. Participants also identified the geography and weather as complicating factors in the decisions about what will happen in their cancer care. , and described by a client: “…sometimes, because of the weather, they [health care providers] want to make sure I won't get stuck up there. They were worried about that last time I came down. They're worried I'm going to get stuck, because they don't have all that hospice stuff back home. Chemotherapy, you can't do that up North”. All participants agreed that the journey to receive cancer care is complex due to medical travel policy, geography, and weather. Those participants who had learned from previous experience described being able to anticipate and participate in decisions on the journey to receive cancer care.

**Theme #3: There is a duty to make decisions that support family and community:**

***“It is important that they are not alone”***

The next theme depicted on the map relates to the experiences of participants on arrival at the urban setting to receive cancer care, and the impacts on their participation in decisions as they navigate the urban setting 1) far from family/community, 2) in a health care setting, and 3) engage in cancer care processes (Figure 1). In our study, participants described how they worked to find ways to collaborate with others in the journey to receive cancer care: “That’s our life, sharing”. Participants described strategies to work with family and community members to negotiate decisions about receiving cancer care in the urban setting and the importance of support to navigate decisions related to cancer care processes, and the impacts on medical escorts who accompany clients.

Participants described how strategies, such as the use of technology can support them to manage complicated family arrangements, and explained by a client: : "My daughter is pregnant and she had to go to [city] [due to the medical evacuation policy] until the due date, and my 13-year old daughter had to follow her sister. Because I didn’t have a sitter right away, in my hometown….Nowadays these [cellphones] are good”. Another client explained how previous life experience had made them aware of the impacts of a family member absence and the importance of maintaining contact: “…when I was a kid my Dad was away for six years in hospital and there was no communication, no telephone, we never – my Mom, myself – heard from him for so long”. The client described the decision to make daily efforts to maintain contact and support for a large and extended family, and the role of technology to facilitate these relationships: “The hardest part was trying to tell my family…But my kids and my relatives say, go for it that’s the best way you will be healed, you will get help. They encouraged me and they tried to have the patience, since we have that text thing, Facebook there, communications day after day, we write each other”. Participants in our study explained how technology allowed them to maintain contact, manage complicated family arrangements, and receive support for decisions related to their cancer care from their family and community members.

The clients also talked about how they rely on support from the medical escorts to help them navigate decisions in their cancer care, while in the urban setting. One client explained the importance of support: "If I don't make a decision, I'll talk to [medical escort] a little bit and [medical escort] lets me decide". Another client explained how they ask the medical escort to help them to engage with health care providers in their cancer care decisions: “I tell my escort, if I get a blank [client forgets something] please ask. That's why I talk to my escort, I ask them previously if I get a blank can you ask them for me”. One client, who had ongoing trouble finding a medical escort, gave an example of the challenge of being alone to manage the decisions involved with navigating cancer care: “So I get there [at the hospital in Ottawa] and I ask the driver where am I supposed to go? I don’t even know which appointment I am supposed to go to in this hospital. She looked at her sheet and said, “It doesn't say. There's an information desk, you go see that person”. And I say, what if she's not there? Because I know which information desk [the driver] was talking about and most of the time somebody is not there? [The driver said] “Then there must be an interpreter, ask them”. I walk in, of course it's [information desk] empty, I walk up to the [OHSNI] interpreters and say hello, I don't even know why I'm here or where I'm supposed to go. So they both start texting their boss at the office."

These client experiences highlight the important role of medical escorts and other supports in negotiating the many day-to-day decisions required to successfully navigate and receive cancer care. For, as one client explained: “I could not have done it by myself, without help”. Clients in our study also reported appreciation for the support from medical escorts who had decided to accompany them on their journey to receive cancer care. Some clients expressed concern about the impacts on medical escorts: “But yesterday…it was very stressful and tiring. It’s difficult for [medical escort ] to be here. I think [medical escort] should be living in housing, for [them] to be here as escort, they’re not making any money for it. They’re not working, so they’re losing money. I think there should be some compensation for that”. When asked directly about the impacts of the decision to be a medical escort, medical escort participants explained that health care for relatives and community members is a priority: “…I’ve got a 7-year-old to look after and last year I was away for the whole year. That was uncomfortable for me because I have to look after [child], because he gets [describes a medical issue]. But I don’t mind if we have to come down to Ottawa, I don’t mind following my [relative], because nobody…wants to follow [relative]”. The medical escorts described their role as a time consuming and demanding job that is important for the wellness of clients: “When they got admitted at the hospital for surgery, I went to visit them every day when they were staying at the hospital - for six, seven, maybe eight hours a day”. All of the medical escorts explained that they independently had to independently learn – or were learning - how to best support clients, and explained by one person: “I try, because I’m here to assist and if I don’t understand anything, how can I assist them?”.

The experiences of participants who are in the urban setting for their cancer care centred on how it is critical that clients receive support and have opportunities to collaborate with others and participate in decisions that make it possible to receive cancer care.

**Theme #4: The lack of knowledge impacts opportunities to engage in decisions during the journey to receive cancer care:**

***"I didn't have a clue about what was going to be happening"***

The final theme relates across the entire journey to receive cancer care, depicted on the map as starting in the home community and through to the urban setting (Figure 1). Participants described the lack of knowledge about cancer care as having an impact on their opportunities to engage in decisions throughout the journey to receive cancer care. One client explained what he wanted to do: “…because having one of those tumors, I wanted to do this, I wanted to do that, but I can’t say because I don’t know what it is. So, there is no way for me to say it. A lot of us don't know the work that doctors do, know what I mean? If I had known that, coming down here would not be too scary, because I was afraid. What is going to happen”.

Some clients explained that they had tried to address their limited understandings of how the cancer care system works as a way to participate in decisions about their cancer care: "When I ask back home, who’s making all these decisions that I have to go to this, or they’re saying you have to be in Ottawa this certain time if I like it or not, even if it interferes with something. And they just tell me, it’s not us, it’s ‘Ottawa’ ". In addition, clients also described the lack of knowledge and the stress of negotiating the cancer care system as leaving them unprepared to participate with health care providers who provide cancer care: "They explain good but when they ask me "any questions?" my mind just gets blank. I don’t know why”. Medical escorts without experience of the cancer care system also described the lack of knowledge as a barrier in their role to support clients. A medical escort described how there was no information about what would happen before they set off from the community to accompany the client to the urban setting for cancer care: “They didn’t explain anything. All they said is you’re going to Ottawa, you have to go”. Even experienced medical escorts reported that they face challenges to support clients in their cancer care: “So, I try to listen to what the doctor or nurse is explaining to the person, what they have to do when they’re being treated, I try to listen to it. But I don’t understand all the medical terminology. It’s a different language. Yes. It’s hard because even when they’re speaking English, it’s like a foreign language to me”. One client related how previous experience with a family member meant that they knew how to participate with health care providers in cancer care decisions: "My [family member] went through cancer care, so I know a little bit about it. I’m more open to ask more questions, so I can get a little bit more answers”. All participants described a lack of knowledge about the cancer care system as a factor that undermines opportunities for Inuit to participate in decisions during the journey to receive cancer care.
